# The Possible Influence of Mediterranean Diet on Extracellular Vesicle miRNA Expression in Breast Cancer Survivors

**DOI:** 10.3390/cancers12061355

**Published:** 2020-05-26

**Authors:** Yu-Jin Kwon, Young-Eun Cho, A-Ra Cho, Won Jun Choi, Sijung Yun, Hyunki Park, Hyung-Suk Kim, Ann K. Cashion, Jessica Gill, Hyangkyu Lee, Ji-Won Lee

**Affiliations:** 1 Department of Family Medicine, Yongin Severance Hospital, Yonsei University College of Medicine, 363 Dongbaekjukjeon-daero, Giheung-gu, Yongin-si, Gyeonggi-do 16995, Korea; digda3@yuhs.ac; 2 National Institute of Nursing Research, National Institutes of Health, 9000 Rockville Pike, Bethesda, MD 20892, USA; young-eun-cho@uiowa.edu (Y.-E.C.); kimhy@mail.nih.gov (H.-S.K.); cashion.ann@gmail.com (A.K.C.); gillj@mail.nih.gov (J.G.); 3 College of Nursing, University of Iowa, 485 CNB 50 Newton Rd., Iowa City, IA 52245, USA; 4 Department of Family Medicine, Yonsei University College of Medicine, Gangnam Severance Hospital 211, Eonju-ro, Gangnam-gu, Seoul 06273, Korea; ARA1713@yuhs.ac (A-R.C.); slashwj@gmail.com (W.J.C.); 5 Yotta Biomed, LLC, 8908 Ewing Dr., Bethesda, MD 20817, USA; sijungyun@yottabiomed.com; 6 College of Nursing, Yonsei University, Mo-Im Kim Nursing Research Institute, 50-1, Yonsei-ro, Seodaemun-gu, Seoul 03722, Korea; maniam@naver.com

**Keywords:** mediterranean diet, breast cancer survivor, microRNA, obesity

## Abstract

The Mediterranean diet (MD) has been reported to have beneficial effects on breast cancer and cardiovascular diseases. Recently, microRNAs (miRNAs) have been suggested as biomarkers for the diagnosis and disease prognosis in cancer and cardiovascular diseases. We evaluated the influence of the MD on the plasma-derived extracellular vesicle miRNA signature of overweight breast cancer survivors. Sixteen participants instructed to adhere to the MD for eight weeks were included in this study. To curate differentially expressed miRNAs after MD intervention, we employed two methods: significance analysis of microarrays and DESeq2. The selected miRNAs were analyzed using ingenuity pathway analysis. After an eight-week intervention, body mass index, waist circumference, fasting glucose, fasting insulin, and homeostatic model assessment for insulin resistance were significantly improved. Expression levels of 798 miRNAs were comprehensively analyzed, and 42 extracellular vesicle miRNAs were significantly differentially regulated after the eight-week MD (36 were up and 6 were down-regulated). We also identified enriched pathways in genes regulated by differentially expressed 42 miRNAs, which include signaling associated with breast cancer, energy metabolism, glucose metabolism, and insulin. Our study indicates that extracellular vesicle miRNAs differentially expressed as a result of the MD might be involved in the mechanisms that relate to cardiometabolic risk factors in overweight breast cancer survivors.

## 1. Introduction

Worldwide, breast cancer is the most commonly diagnosed cancer and leading cause of female cancer death [1]. Recent advances in treatment and improvements in early detection and screening for this disease continue to increase the number of breast cancer survivors [2]. Given the decline of the mortality rate of breast cancer patients [2], survivorship care after breast cancer is an important issue not only for oncologists but also for general practitioners [3]. Obesity, physical inactivity, and unhealthy diet are known to increase mortality of breast cancer survivors [4,5]. Because breast cancer and cardiovascular diseases (CVD) share common risk factors, the risk of CVD is higher in older breast cancer survivors [6]. Therefore, weight management, sufficient physical activity, and health diet pattern are crucial for decreasing cancer mortality and CVD among breast cancer survivors [6,7]. The Mediterranean diet (MD) is characterized by a high intake of vegetables, fruits, nuts, legumes, whole grains, and olive oil, as well as a moderate consumption of fish and poultry, and a low intake of sweets and red meat [8,9]. MD beneficial effects in metabolic diseases, such as CVD, various cancers, and obesity have been extensively documented over the past decade [8,10,11]. In addition, several studies have reported that the MD had a primary prevention effect in breast cancer [12,13,14,15]. Toledo et al. [12] found that a MD supplemented with extra virgin olive oil reduced the risk of breast cancer in the Prevención con Dieta Mediterránea (PREDIMED) trial. The Singapore Chinese Health Study of an Asian cohort showed that the MD prevented the incidence of breast cancer in overall population and postmenopausal women [15]. The California Teachers Study demonstrated that a high index of MD was associated with a low risk of breast cancer incidence in postmenopausal women [14].

The extracellular vesicle (EV), a membrane-derived vesicle, is involved in cell-to-cell communication and contains various molecules, such as lipids, mRNAs, and microRNAs (miRNAs) [16]. EV miRNA has sufficient properties to be a useful biomarker which is detected preferably before the clinical symptoms have appeared [17]. Previous studies have shown alteration of miRNAs after a relatively short duration of diet intervention and their associations with metabolic parameters [18,19]. Ortega et al. found circulating miRNA profile changes from 30 participants who consumed nuts (Polyunsaturated fatty acids (PUFAs)-enriched diet) for 8 weeks [18]. Marques-Rocha et al. reported that expression levels of inflammation-related miRNAs were changed after MD-based weight loss program in individuals with metabolic syndrome [19]. However, we still have not fully understood the mechanisms that change the miRNA expressions after MD intervention in human. Previous studies have analyzed only a small number of candidate genes curated from the knowledge-based or local biological function-based miRNA pools [19]. Therefore, in this study, we aimed to evaluate the influence of the MD on cardiometabolic changes and EV miRNA signature in breast cancer survivors by screening more than 700 significant miRNAs.

## 2. Methods

### 2.1. Study Overview and Participants

The original randomized controlled trial “Effects of Mediterranean diet and naltrexone/bupropion treatment on weight loss in overweight or obese breast cancer survivors” (ClinicalTrials.gov number, NCT03581630) was designed as three arms; the first group was overweight breast cancer survivors receiving the MD plus naltrexone/bupropion medication; the second group was overweight breast cancer survivors receiving the MD alone; and the third group was overweight non-cancer patients receiving the Mediterranean diet plus naltrexone/bupropion medication. To rule out the effect of the drug, we included twenty overweight breast cancer survivors who received the MD alone into the sub-study of EV miRNA expression before and after the intervention. All participants agreed to the measurements of the circulating EV miRNA in their samples.

Inclusion criteria were as follows: breast cancer survivors who were diagnosed with breast cancer stage Ⅰ–Ⅲ and who completed any cancer treatment at least 5 years ago, aged 20–65 years, obese (BMI ≥ 25 kg/m^2^), or overweight 25 kg/m^2^ ≥ BMI ≥ 23 kg/m^2^ with at least one metabolic risk factor with Asian criteria. The participants who experienced cancer recurrence or metastasis were excluded. The MD intervention was performed for 8 weeks. The participants received dietary advice regarding the MD from a qualified nutritionist during a 30 min baseline visit. The subjects also received daily feedback about their food consumption from the researchers through a mobile messenger. Before and after MD intervention, the degree of adherence to the MD was measured using the modified 13-item questionnaire Mediterranean diet screener (excluding red wine consumption) developed by the PREDIMED study group [8,20]. Samples from four participants failed quality control procedures; we conducted full analysis of data from 16 out of 20 participants enrolled initially. This study was conducted at the Gangnam Severance Hospital (Seoul, South Korea) according to the principles of the International Declaration of Helsinki. The study was approved by the institutional review board (3-2017-0097) and written informed consents were obtained from all participants before the trial. 

### 2.2. Extracellular Vesicle RNAs Measurement

**Figure A1:**
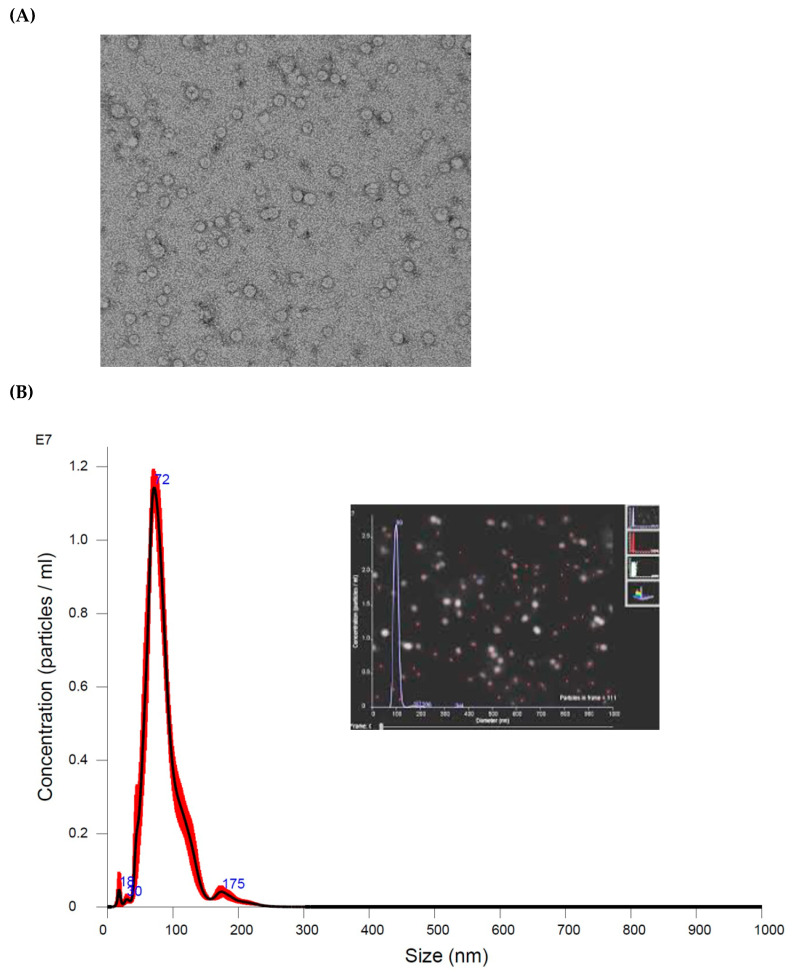
Characteristics of plasma extracellular vesicles (EVs). (**A**) Extracted plasma EVs were scanned by electron-microscopy. (**B**) Size and concentration of EVs were measured by NanoSight 300.

**Figure A2:**
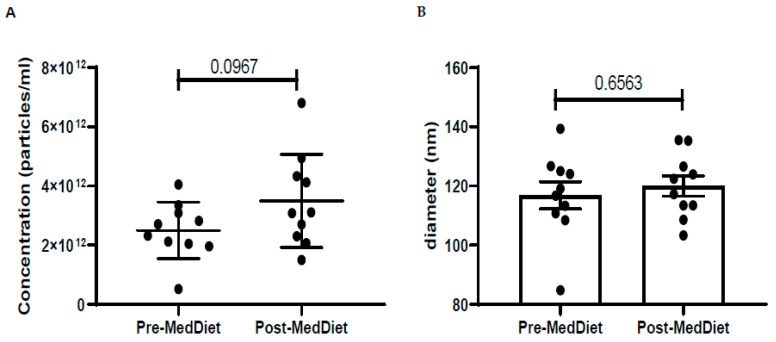
Effect of Mediterranean Diet on extracellular vesicles characteristics. (**A**) Concentration of plasma EVs between pre-MD and post-MD were compared, (**B**) Size of plasma EVs between pre- MD and post- MD were compared.

EV RNA was extracted from 1.5 mL of plasma by using an exoRNeasy Serum/Plasma kit (Qiagen, Hilden, Germany) according to the manufacturer’s instruction. Exogenous spike-in cel-miR-39-3p was added to each sample during the extraction as loading control. After isolation, samples were cleaned and concentrated (Zymo Research, Irvine, CA, USA), then concentrations were measured with Qubit (Thermo Fisher Scientific, Waltham, MA, USA). All samples were loaded into a NanoString human miRNA panel (NanoString, Seattle, WA, USA) to measure the miRNA expression at pre-and post- MD intervention. miRNA expression level of each sample was normalized with the expression value of spike-in and housekeeping genes on nSolverTM (NanoString, Seattle, WA, USA). We presented the characteristics of plasma EVs in Appendix A Figure A1 and effects of MD on EVs characteristics in Appendix A Figure A2. The sizes and concentrations of EVs were not significant in both groups.

### 2.3. Identification of Differentially Expressed miRNAs

In order to curate differentially expressed miRNAs after MD intervention, we employed two methods: significance analysis of microarrays (SAM) and DESeq2. The SAM approach proposed by Tusher et al. [21] estimates false discovery rate (FDR) by applying the permutation method. We used the “SAM” function with setting “Two class paired” and “nperms (number of permutation) = 500” in samr package [21]. DESeq2 uses shrinkage estimation for dispersions and fold changes to improve stability and interpretability of estimates [22]. We used the “DESeq()” function in the DESeq2 package [22]. For both SAM and DESeq2, we set the adjusted *p*-value (FDR) to be below 0.05 and |log2(fold change)| to be over 1 as the cut-offs for differentially expressed miRNAs.

Given that we analyzed relatively few samples (*n* = 16), even one sample with outliers could over- or underestimate the effects of the MD intervention. We observed that 14 miRNAs at baseline point had standard deviation values over 100, indicating that there were outliers that could indeed mask the significant effects. To overcome the limitation of the relatively small sample size, we performed bootstrapping with the two described methods, SAM and DESeq2, as follows:

Step (1): Sixteen samples (same number in all subjects) were randomly selected with replacement.
Step (2): Differentially expressed miRNAs were curated by using SAM and DESeq2 with adjusted *p*-value (FDR) < 0.05 and |log2(fold change)| > 1.
Step (3): The above steps were repeated 100 times, yielding a list that included selected miRNAs.

### 2.4. Curation of miRNA Target Genes

The selected miRNAs were analyzed by ingenuity pathway analysis (IPA; QIAGEN, Hilden, Germany). Putative miRNA target genes were filtered based on the setting that included only experimentally validated targets. For miRNA that targeted too many genes (>50), stricter criteria (experimentally validated + highly confident prediction) were applied.

### 2.5. Pathway Analysis

We performed pathway analysis for candidate genes by using Kyoto Encyclopedia of Genes and Genomes (KEGG) [23] and Gene Ontology (GO) [24] databases and the DAVID tool (https://david.ncifcrf.gov/) [25]. A pathway was deemed to be significantly enriched in genes targeted by miRNAs if FDR < 0.05.

## 3. Results

### 3.1. Clinical Characteristics of the Study Population

The Severance Mediterranean Diet team (SeMeD) performed the dietary intervention for breast cancer survivors, and 11 cardiometabolic indices were measured (Table 1).

**Figure 1:**
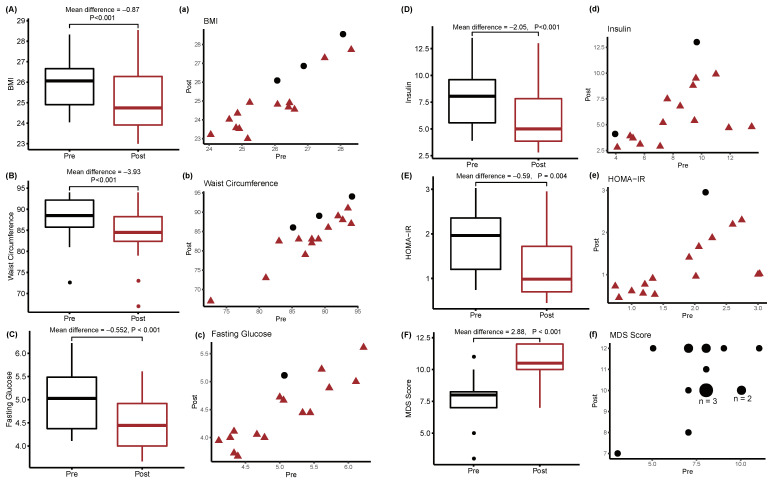
Changes in metabolic indices induced by the MD in overweight breast cancer survivors. The panels annotated by the capital letters (**A**–**F**) represent box-whisker plots of metabolic indices before and after intervention. *p*-values were calculated by the paired Student’s *t*-test after Kolmogorov–Smirnov test. The panels annotated by the lowercase letters (**a**–**f**) represent scatter plots of the same data as in (**A**–**F**). Red triangles indicate values that decreased after the intervention. Black circles indicate values that increased after the intervention.

After an eight-week intervention, body mass index (BMI), waist circumference (WC), fasting glucose, fasting insulin, and homeostatic model assessment for insulin resistance (HOMA-IR) were significantly reduced (Figure 1A–E). In particular, 13 of the 16 participants had lower BMI and smaller WC after the eight-week MD intervention. Furthermore, 14 of the 16 participants had lower insulin levels, whereas 15 of the 16 participants showed decreased levels of fasting glucose and HOMA-IR. The Mediterranean Diet Screener (MDS) score (Figure 1F) was significantly improved after 8 weeks of the intervention (7.8 ± 1.9 before MD and 10.7 ± 1.5 after MD, *p* < 0.001), indicating improved adherence to MD in all participants.

### 3.2. Plasma Extracellular Vesicle miRNA Changes and Mediterranean Diet

First, extracted extracellular vesicles were confirmed by an electron microscope and Nanoparticle Tracking Analysis (NTA) (Appendix A Figure A1). NTA showed that the average size and concentration of extracellular vesicles were not significantly different between pre-and post-MD (Appendix A Figure A2). 

We screened 798 miRNAs and measured its fold changes between pre- and post-MD intervention in 16 subjects (798 × 16 matrix). We applied two linear regression models with one setting each miRNA as the dependent variable and WC as the independent variable, and the other treating “WC + MDS” as the independent variable. We determined two sets of 798 R2 values, which reflected the fractions of total variation in expression of 798 miRNAs (dependent variable) that could be explained by WC and “WC + MDS” annotations. Similar analyses were also performed for the effects of BMI and HOMA-IR with and without MDS scores.

**Figure 2:**
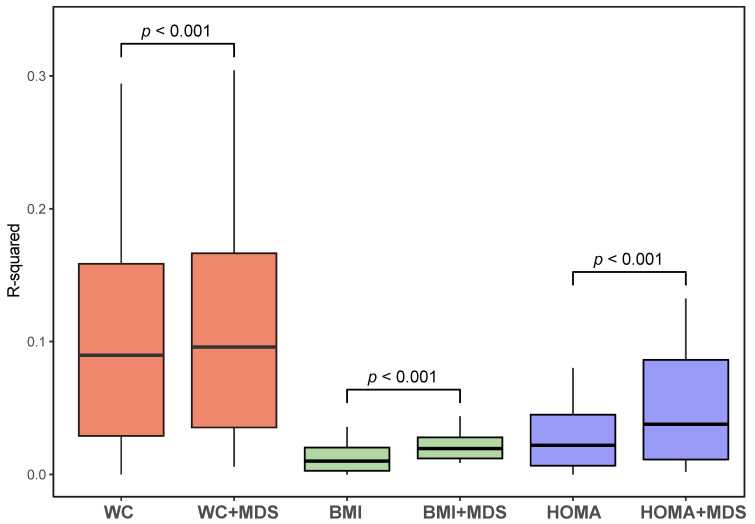
Proportions of variance in miRNA expression (R2) explained by WC, BMI, and HOMA-IR respectively and in combination with MDS scores. MiRNA, micro RNA; WC, waist circumference; BMI, body mass index; HOMA-IR, homeostasis model assessment of insulin resistance; and MDS, Mediterranean diet screener.

Figure 2 shows R2 values for the effects of WC, BMI, and HOMA-IR with and without MDS scores. The combined effects of WC, BMI, HOMA-IR, and MDS scores on miRNA changes were significantly higher than the effects of WC, BMI, and HOMA-IR without MDS scores (all *p*-values < 0.001), respectively. These results implied that MD had a crucial contribution to transcriptomic alterations independent of WC, BMI, or HOMA-IR in breast cancer survivors.

### 3.3. Identification of Expressed Extracellular Vesicle miRNAs

**Figure 3:**
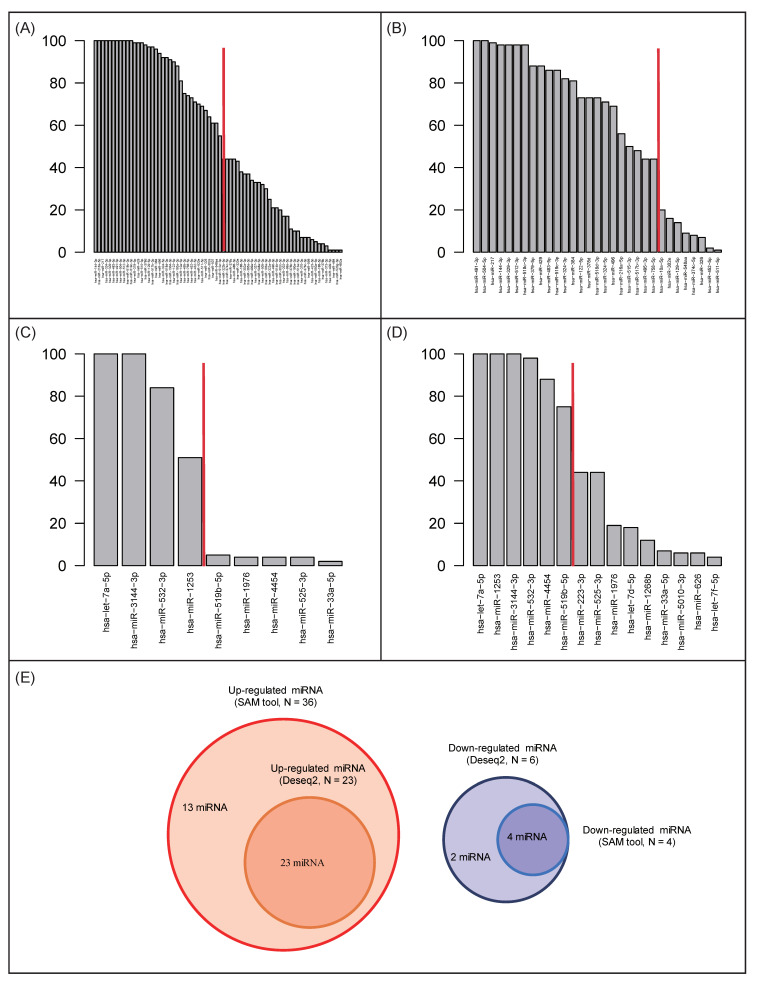
Differentially regulated miRNAs after MD identified using SAM and DESeq2. (**A**) Up-regulated miRNAs identified using SAM. (**B**) Up-regulated miRNAs identified using DESeq2. (**C**) Down-regulated miRNA identified using SAM. (**D**) Down-regulated miRNAs identified using DESeq2. (**E**) Numbers of miRNAs selected using both SAM and DESeq2.

Figure 3 shows up- or downregulated miRNAs selected by the “Significance Analysis of Microarrays” (SAM) tool with bootstrapping. The 70 miRNAs upregulated by MD were selected with different frequencies; for example, hsa-miR-144-3p was selected 100 times, whereas hsa-miR-892a was selected only once (Figure 3A). In the same way, 9 downregulated miRNAs were selected (Figure 3C). By using DESeq2 with bootstrapping, 31 upregulated and 15 downregulated miRNAs were selected, respectively (Figure 3B,D).

The rapidly decreasing point for the 70 upregulated miRNAs selected using SAM was at the frequency value of 51 (Figure 3A). In the same way, we determined frequency values of 44 and 71 as thresholds for upregulated miRNAs (DESeq2) and downregulated miRNAs (DESeq2), respectively (Figure 3B,D). Finally, 36 and 23 up-regulated miRNAs, as well as four and six downregulated miRNAs, identified using SAM and DESeq2, respectively, were selected (Figure 3E). The list of 36 up-regulated miRNAs selected using SAM included all 23 miRNAs from the DESeq2 list. Furthermore, the six downregulated miRNAs identified using DESeq2 included all miRNAs selected using SAM. Detailed information about the selected miRNAs is presented in Table S1.

### 3.4. Extracellular Vesicle miRNAs and Their Target Genes

To identify the genes targeted by the selected miRNAs, we used QIAGEN Ingenuity Pathway Analysis (IPA) software, taking into account only experimentally validated entries. As a result, 240 genes were identified as target genes of the 14 miRNAs that were altered by the MD (Table 2).

**Figure 4:**
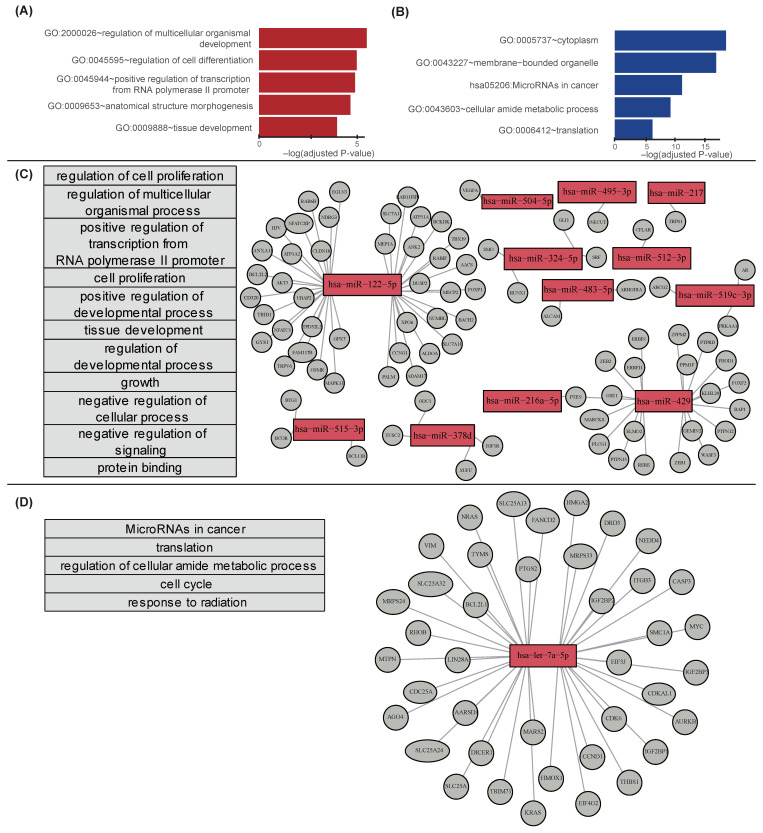
Top five pathways from annotations of target genes of differentially regulated miRNAs after MD. (**A**) Top five pathways of the genes targeted by the up-regulated microRNAs, and (**B**) down-regulated microRNAs. Horizontal bars in (**A**,**B**) indicate −log (*p*-value) calculated using IPA. (**C**) Target genes regulated by the up-regulated microRNAs. (**D**) Target genes regulated by the down-regulated microRNA hsa-miR-let-7a-5p.

In the pathway analysis, 99 genes targeted by 13 up-regulated miRNAs were enriched in 19 GO pathways (Table S2). Figure 4A shows top five pathways involving genes targeted by up-regulated miRNAs. The 131 genes targeted by one down-regulated miRNA were enriched in 27 GO and 2 KEGG pathways (Table S3). Figure 4B shows top five pathways of the genes targeted by down-regulated miRNAs (cytoplasm, membrane bounded organelle, microRNAs in cancer, cellular amide metabolic process, and translation).

Among the 48 pathways (19 + 29 pathways) overrepresented in sets of potential target genes, we identified breast cancer-related pathways via literature search (Figure 4C,D). The 19 pathways enriched in the genes targeted by up-regulated miRNAs were associated with multidrug resistance, triple-negative breast cancer, and tamoxifen-induced estrogen receptor α functions (Table S2). The 29 pathways enriched in the genes targeted by the down-regulated miRNAs were reported to be associated with lapatinib-resistant breast cancer, triple-negative breast cancer, and invasive ductal carcinoma (Table S3).

We curated genes related to obesity (*n* = 201), inflammation (*n* = 2229), insulin resistance (IR, *n* = 175), and breast cancer (*n* = 3141) from the DigSee database [26]. Among the 240 genes targeted by 14 altered miRNAs, 45 genes targeted by nine altered miRNAs were common with inflammation-related genes from DigSee. The 5, 7, and 80 genes targeted by 2, 5, and 12 miRNAs were common with IR-, obesity-, and BRCA-related genes, respectively. The detailed information about these genes is provided in Table S4.

## 4. Discussion

In our study, we found that 16 overweight breast cancer survivors who were instructed to adhere to the MD had significantly lower BMI, WC, fasting glucose, and HOMA-IR after 8 weeks on this diet. In addition, despite the relatively short intervention time, the MD altered expression of tens of miRNAs among the 798 miRNAs analyzed in our experiments. Differentially regulated miRNAs after MD intervention are known to be related to obesity, inflammation, and insulin resistance.

Although the overall breast cancer mortality rate has been consistently decreasing, breast cancer is still the most common cause of cancer death among women, and 30% of all women initially diagnosed with early-stage breast cancer experience relapses at distant sites [27]. This risk is not only reflected by the molecular cancer subtype, TNM stage, and cancer grade, but also by the tumor cell genetics [27]. To evaluate the risk for cancer recurrence more precisely, a number of non-invasive surveillance options have been explored, ranging from diverse imaging tools to the analysis of body fluids with the aim of detecting circulating tumor cells, tumor DNA, proteins, miRNAs, and extracellular vesicles [28,29].

Recently, there has been a growing interest in circulating miRNAs as cancer biomarkers due to their stability and noninvasiveness [30]. miRNAs are noncoding RNAs that regulate the expression of target genes at the posttranscriptional level and play crucial roles in cellular processes such as cell differentiation, proliferation, and apoptosis [31]. In breast cancer, a number of miRNAs have been identified as tumor suppressors or oncogenes, which have important effects on tumor initiation, metastasis, and chemoresistance [30]. These results suggest that determination of certain miRNAs may facilitate the diagnosis, disease prognosis, and treatment response predictions in breast cancer. Besides unfavorable tumor characteristics, individual factors such as diet, exercise, and pre-existing medical conditions also affect cancer recurrence and metastasis [32]. Obesity, chronic inflammation, and insulin resistance are modifiable risk factors that contribute to the development of breast cancer occurrence [32,33,34,35]. Controlling weight, exercising, and healthy eating could help lowering the risk of breast cancer recurrence as well as protect from other chronic diseases, including CVD.

Accumulating evidence suggests that adherence to MD reduces the risk of breast cancer as well as of obesity, metabolic syndrome, and CVD [12,13,36,37]. Anti-inflammatory and antioxidant effects of MD conferred by extra virgin olive oil and high amounts of fiber and polyphenols have been regarded as protective factors against breast cancer [19,38]. A meta-analysis of randomized controlled trials revealed that MD consumption was associated with significant weight loss (mean difference, −1.75 kg; 95% confidence interval: −2.86 to −0.64 kg) and lower BMI (mean difference, −0.57 kg/m², −0.93 to −0.21 kg/m²) [36]. A recent PREDIMED-Plus study showed that MD with exercise for 1 year was effective in decreasing cardiovascular risk factors, including WC, glucose, and insulin resistance (mean differences −3.1 cm, −0.23 mmol/L, and −1.16) in overweight/obese older adults [37]. Similarly, BMI and WC were significantly lower in the current study after an 8-week period on MD (mean differences: −0.87 and −3.93 cm), respectively. The levels of glucose and HOMA-IR were also significantly decreased by MD (mean differences, −0.55 mmol/L and −0.59, respectively), which were comparable to the changes seen after 1 year in the PREDIMED study.

A recent study revealed that Mediterranean-based nutritional intervention induced changes in miRNAs expression levels [19]. Moreover, several studies have highlighted the significance of miRNAs as potential therapeutic targets for obesity and metabolic disorders [39,40]. Thus, we hypothesized that even short adherence to the MD pattern potentially affects the expression of miRNAs related to cancer recurrence as well as provides metabolic benefits in overweight breast cancer survivors. Most previous studies analyzed few candidate miRNAs curated using knowledge-based selection (i.e., specific PCR primers/probes), a strategy that could lead to biased information. In contrast, we comprehensively analyzed a total of 798 miRNAs and revealed 36 and 6 miRNAs up- and downregulated, respectively, after an 8-week MD. Although we screened miRNAs using Nanostring technology which detects almost 800 selected miRNAs from total miRNAs (>2000), untested miRNA might be involved in the effect of the MD. In addition, when MDS score was combined with WC, BMI, or HOMA-IR, the explanatory power of these annotations on miRNA changes was significantly elevated in terms of R2 values. These results could suggest that adherence to MD, per se, causes beneficial miRNA changes in breast cancer survivors.

In this study, EV miRNAs involved in breast cancer invasion and metastasis by regulating multiple cellular processes were differentially regulated after MD intervention. Hsa-miR-122-5p was reported to suppress syndecan-1 expression, inhibiting breast cancer cell mobility [41]. Hsa-miR-324-5p was demonstrated to have different patterns of expression, depending on breast cancer type, and the overexpression of this miRNA suppressed cancer cell growth and invasion [42]. Let-7 miRNA is usually categorized as a tumor suppressor because it inhibits migration and invasion, as well as epithelial-mesenchymal transition by regulating several oncogenes [43]. However, the role of let-7 as a tumor suppressor is controversial, and emerging data suggest that, counterintuitively, in some situations, let-7 may act as an oncogene, increasing cancer migration, invasion, and expression of genes associated with progression and metastasis [44]. Interestingly, in our analysis, hsa-let-7a-5p was downregulated after the MD. These inconsistent signatures of let-7 can be attributed to the differences in the study populations and methodologies used in different reports. Further studies are warranted to explore let-7 targets and biological context-specific flexibility of let-7 functions.

EV miRNAs associated with energy metabolism, glucose metabolism, and insulin signaling were also altered. Hsa-miR-329-3p was reported to be downregulated in obese hyperglycemic mice [45], and hsa-miR-512-3p was shown to be involved in adipocyte browning [46]. DNA methylation of CpG sites located in hsa-miR-216a-5p is altered in obese children [47]. Both hsa-miR-324-3p and hsa-miR-324-5p, which were obtained from visceral adipose tissue, were also up-regulated after MD intervention in our study and down-regulated in non-alcoholic fatty liver disease [48]. Targets of dysregulated let-7 are known to contribute to glucose homeostasis and insulin signaling as well as cancer pathophysiology [49,50,51]. However, the roles of let-7 functionally relevant targets in metabolic control await further elucidation. Hsa-let-7a-5p is down-regulated in individuals with diabetes [50], whereas pancreas-specific overexpression of let-7 in mice resulted in impaired glucose tolerance and reduced pancreatic insulin secretion [49]. In contrast, we observed that hsa-let-7a-5p was downregulated after MD, whereas glucose control and insulin resistance were improved. Global knockdown of the let-7 family prevented impaired glucose tolerance and insulin resistance in obese mice [49], which agrees with our findings. Therefore, further studies are required to clarify the exact physiological and pathological roles of let-7 in aberrant metabolic processes.

The target genes of the 14 miRNAs altered by the MD were predicted using QIAGEN IPA software and DigSee database. Among the 240 target genes selected by IPA, 80 breast cancer-related, 7 obesity-related, 5 IR-related, and 45 inflammation-related genes overlapped with those predicted from the DigSee database. Among breast cancer-, inflammation-, and IR-related genes, the biological events were “Gene expression”, “Mutation”, “Positive or Negative regulation”, and “Mutation”. In obesity-related genes, the biological events were “Mutation”, “Gene expression”, “Positive regulation”, and “Localization”. Due to the complex regulation of the target genes, more studies are needed to verify the exact roles of various genes in relation to miRNA changes in breast cancer pathophysiology.

To determine biological processes and molecular functions associated with miRNAs differentially expressed as a result of the MD, we performed pathway analysis and identified several potentially affected pathways related to breast cancer pathogenesis. We found several such pathways enriched in genes targeted by upregulated miRNAs, which was consistent with previous studies; for example, the “regulation of developmental process (GO:0050793)” pathway was the key candidate pathway related to breast cancer in Han Chinese women [52]. The “growth (GO:0040007)” and “regulation of cell proliferation (GO:0042127)” pathways were reported to be related to triple-negative breast cancer [53]. “Tissue development pathway (GO:0009888)” and “multicellular organismal development pathway (GO:2000026)” were associated with genes altered in invasive breast cancer [54] and multidrug resistance in MCF-7/MDR breast cancer cells [55]. Most pathways enriched in annotations of genes targeted by downregulated miRNAs were associated with the pathogenesis and progression of breast cancer: microRNAs in cancer [hsa05206] [56], translation [GO:0006412] [57], reproductive system development [GO:0061458] [58], cell cycle [GO:0007049] [58], adherens junction [GO:0005912], and anchoring junction [GO:0070161]) [59]. Although it is impossible to understand fully all biological phenomena driving gene expression and phenotypic changes, our approach has a potential clinical applicability and paves the way for future research on breast cancer prognosis.

Our study had several limitations. First, the weakness of this study was a relatively small sample size without control group. This is a pilot study that used the subset of the following trial (NCT 03581630). Currently, larger randomized controlled trials with two arms (breast cancer patients taking the MD and those taking conventional diet) are ongoing. Second, the intervention period to assess the effects of the MD on metabolic risk was short. However, we found significant beneficial effects on metabolic parameters despite 8 weeks of MD intervention. Changes in the molecular level support our findings. Third, due to the shortage of the sample for the further analysis, we could not validate the expression levels of target gene and determine the directions of effect of those miRNAs on target genes. Further confirmation of target gene expressions will be necessary. However, we observed transcriptomic alterations, specifically, the miRNA signatures, indicating that the perturbed or re-perturbed genetic changes in the form of miRNA molecules precede phenotypic changes in breast cancer survivors [60]. To establish the direction of changes in target genes following alterations in miRNA levels, the multi-omics approach, including transcriptomics and proteomics, will be needed in the future.

## 5. Conclusions

We found that 8 weeks of the MD causes changes in the expression of EV miRNAs in overweight breast cancer survivors. Differentially regulated miRNAs after MD intervention were related to obesity, inflammation, and insulin resistance. Further exploration of the downstream of EV miRNA might provide novel targets for preventing cancer recurrence in overweight breast cancer

## Figures and Tables

**Table 1:** Baseline characteristics of study participants (*n* = 16).

Body Composition	Patients’ Data	Nutritional Status	Patients’ Data
Age (Years)	55.2 ± 7.2	Total energy intake(kcal)	1646 ± 411
SBP (mmHg)	126.4 ± 13.4	Carbohydrate (%)	51.4 ± 14.7
DBP (mmHg)	83.8 ± 11.9	Protein (%)	16.3 ± 2.0
Hear rate (bpm)	75.0 (68.3, 80.8)	Fat (%)	28.8 ± 10.6
**Metabolic parameters**		SFA (%)	5.2 (2.1, 7.3)
BMI (kg/m^2^)	26.0 ± 1.3	MUFA (%)	6.1 (4.1, 8.7)
Waist circumference(cm)	87.8 ± 5.6	PUFA (%)	8.7 (5.0, 14.8)
Skeletal muscle (kg)	22.2 (21.5, 22.9)	Omega-6 (%)	0.5 (0.1, 1.6)
Fat mass (kg)	23.4 ± 3.5	Omega-3 (%)	2.2 (0.8, 9.3)
Fat percentage (%)	36.3 ± 4.3	**Hormone status**	
**GLTEQ score** (MET-h/week)	9.3 (0, 28)	ER−/PR−	4 (25%)
Glucose (mg/dL)	90.1 ± 12.0	ER+/PR−	2 (12.5%)
Insulin (mU/L)	8.1 ± 2.8	ER−/PR+	1 (6.25%)
HOMA-IR	1.8 ± 0.8	ER+/PR+	9 (56.25%)
Total cholesterol (mg/dL)	198.0 ± 26.3	**Staging**	
Triglyceride (mg/dL)	148.4 ± 67.6	IA	9(56.3%)
HDL cholesterol (mg/dL)	56.0 ± 9.9	IIA	4(25%)
LDL cholesterol (mg/dL)	112.3 ± 24.3	IIB	3 (18.7%)

BMI, body mass index; SBP, systolic blood pressure; DBP, diastolic blood pressure; HOMA-IR, homeostasis model assessment of insulin resistance; HDL, high density lipoprotein; LDL, low density lipoprotein; GLTEQ, Godin Leisure-Time Exercise Questionnaire; SFA, saturated fatty acid; MUFA, monounsaturated fatty acid; PUFA, polyunsaturated fatty acid; ER, estrogen receptor; and PR, progesterone receptor. Data are presented as mean ± standard deviations for continuous variables with normal distribution or median (25th, 75th) for continuous variables with markedly skewed distribution or number (percentage) for categorical variables.

**Table 2:** Changed miRNAs and target genes by ingenuity pathway analysis (IPA).

MicroRNAs	Log_2_(FC)	*p* Value	Putative Target Genes by IPA
hsa-miR-122-5p	1.095	<0.001	AACS, ADAM17, AKT3, ALDOA, ANK2, ANXA11, AP3M2, ATP11A, ATP1A2, BACH2, BCKDK, BCL2L2, CCNG1, CD320, CERS6, CLDN18, CS, DSTYK, DUSP2, EGLN3, ENTPD4, FAM117B, FOXJ3, FOXP1, FUNDC2, G6PC3, GALNT10, GPX7, GYS1, HJV, MAPK11, MECP2, MEP1A, NCAM1, NDRG3, NFATC1, NFATC2IP, NUMBL, OSMR, PALM, RAB11FIP1, RAB6B, RABIF, SLC35A4, SLC7A1, SLC7A11, TBX19, TMED3, TMEM50B, TPD52L2, TRIB1, TRPV6, TTYH3, UBAP2, XPO6
hsa-miR-429	1.145	<0.001	BAP1, ELMO2, ERBIN, ERRFI1, FHOD1, FOXF2, GEMIN2, GSE1, KLHL20, MARCKS, PLCG1, PPM1F, PTEN, PTPN12, PTPN13, PTPRD, RERE, WASF3, WDR37, ZEB1, ZEB2, ZFPM2
hsa-miR-324-5p	1.003	<0.001	GLI1, RUNX1, SMO, SRF
hsa-miR-378d	0.933	<0.001	IGF1R, ODC1, SUFU, TUSC2
hsa-miR-483-5p	1.019	<0.001	ALCAM, ARHGDIA
hsa-miR-515-3p	0.89	<0.001	BCL11B, BCOR, BTG1
hsa-miR-519c-3p	1.007	<0.001	ABCG2, AR, PRKAA1
hsa-miR-144-3p	1.359	<0.001	ENPP6
hsa-miR-216a-5p	0.996	<0.001	PTEN
hsa-miR-217	1.308	<0.001	TRPS1
hsa-miR-495-3p	0.891	<0.001	ONECUT1
hsa-miR-504-5p	1.207	<0.001	VEGFA
hsa-miR-512-3p	1.403	<0.001	CFLAR
hsa-let-7a-5p	−3.794	<0.001	AARSD1, ACP1, ADGRG1, AGO4, AKAP8, ANAPC1, ATAD3B, ATP6V0A1, ATP6V1F, AURKB, BCL2L1, BCL7A, BMP2K, BSG, CALCOCO2, CAPG, CARHSP1, CASP3, CCND1, CDC25A, CDIPT, CDK6, CDKAL1, CEMIP2, CHMP2A, CIAO2A, COIL, COL1A2, COMMD9, CSDE1, CSNK1D, DAD1, DHX57, DICER1, DOCK5, DRD3, DSP, DUSP12, DUSP23, EIF3J, EIF4G2, F2, FADS2, FANCD2, FNDC3A, GAK, GEMIN7, GRPEL2, GTPBP3, GYS1, HMGA1, HMGA2, Hmga2, HMOX1, HYOU1, IFIT5, IFRD1, IGF2BP1, IGF2BP2, IGF2BP3, IPO4, ITGB3, KCNJ16, KLK10, KRAS, KRT19, LIN28A, MARS2, MED28, MLLT1, MRM1, MRPS24, MRPS33, MTPN, MTRR, MYC, NEDD4, NF2, NRAS, NXN, OTULINL, PGRMC1, POLD2, POLR2C, POM121/POM121C, PPP1R7, PRDM1, PRIM1, PRRC2A, PTGS2, PXDN, RABGAP1L, RAS, RBM19, RDH10, RHOB, RHOG, RPP38, RRP8, RTCA, SCYL1, SEPT3, SIGMAR1, SLC1A4, SLC25A1, SLC25A13, SLC25A24, SLC25A32, SLC38A1, SMC1A, SMOX, SNAP23, SPCS3, SPRYD4, SYPL1, TAF9B, TAGLN, TGFBR1, THBS1, TLR4, TPM2, TRIM71, TRMT1, TTC9C, TUSC2, TYMS, UGT8, UHRF1, VIM, VPS39, WNT1

## Supplementary Materials

The following are available online at https://www.mdpi.com/2072-6694/12/6/1355/s1, Table S1: Selected miRNAs by SAM and DEseq2 tool; Table S2: Up-regulated pathway and target genes; Table S3: Down-regulated pathway and target genes, Table S4: Detailed information about IR-, obesity-, and BRCA-related genes.
