# Supplementary material for: The Possible Influence of Mediterranean Diet on Extracellular Vesicle miRNA Expression in Breast Cancer Survivors
Source: Cancers (Basel). 2020 May 26;12(6):1355. doi: 10.3390/cancers12061355 (PMC7352167; doi:10.3390/cancers12061355)
Supplement: Supplementary file 1 [file cancers-12-01355-s001.zip › Table S1.docx]

**Supplementary Table S1. Selected miRNAs by SAM and DEseq2 tool**

| miRNAs | SAM | DEseq2 | log2FoldChange | Adjusted P value (FDR) |
| --- | --- | --- | --- | --- |
| hsa-miR-122-5p | selected as up-regulated miRNAs | selected as up-regulated miRNAs | 1.094822636 | 0.000743998 |
| hsa-miR-144-3p | selected as up-regulated miRNAs | selected as up-regulated miRNAs | 1.358956569 | 1.03E-11 |
| hsa-miR-216a-5p | selected as up-regulated miRNAs | selected as up-regulated miRNAs | 0.996400677 | 9.87E-06 |
| hsa-miR-217 | selected as up-regulated miRNAs | selected as up-regulated miRNAs | 1.308296163 | 1.29E-07 |
| hsa-miR-324-3p | selected as up-regulated miRNAs | selected as up-regulated miRNAs | 0.960672983 | 1.50E-09 |
| hsa-miR-324-5p | selected as up-regulated miRNAs | selected as up-regulated miRNAs | 1.002567233 | 1.32E-05 |
| hsa-miR-329-3p | selected as up-regulated miRNAs | selected as up-regulated miRNAs | 1.174931486 | 4.95E-07 |
| hsa-miR-378d | selected as up-regulated miRNAs | selected as up-regulated miRNAs | 0.932974553 | 5.63E-06 |
| hsa-miR-379-5p | selected as up-regulated miRNAs | selected as up-regulated miRNAs | 1.19358449 | 8.93E-09 |
| hsa-miR-384 | selected as up-regulated miRNAs | selected as up-regulated miRNAs | 1.146547158 | 0.000815139 |
| hsa-miR-429 | selected as up-regulated miRNAs | selected as up-regulated miRNAs | 1.144981858 | 1.08E-05 |
| hsa-miR-483-5p | selected as up-regulated miRNAs | selected as up-regulated miRNAs | 1.018994706 | 3.62E-05 |
| hsa-miR-491-3p | selected as up-regulated miRNAs | selected as up-regulated miRNAs | 1.388541623 | 9.93E-10 |
| hsa-miR-495-3p | selected as up-regulated miRNAs | selected as up-regulated miRNAs | 0.89117221 | 0.000394935 |
| hsa-miR-496 | selected as up-regulated miRNAs | selected as up-regulated miRNAs | 0.902762775 | 5.91E-05 |
| hsa-miR-504-5p | selected as up-regulated miRNAs | selected as up-regulated miRNAs | 1.207031825 | 6.42E-07 |
| hsa-miR-512-3p | selected as up-regulated miRNAs | selected as up-regulated miRNAs | 1.402704134 | 2.32E-05 |
| hsa-miR-515-3p | selected as up-regulated miRNAs | selected as up-regulated miRNAs | 0.889688366 | 6.57E-05 |
| hsa-miR-517b-3p | selected as up-regulated miRNAs | selected as up-regulated miRNAs | 0.836150072 | 0.000817146 |
| hsa-miR-518c-3p | selected as up-regulated miRNAs | selected as up-regulated miRNAs | 1.233075103 | 1.66E-07 |
| hsa-miR-518d-3p | selected as up-regulated miRNAs | selected as up-regulated miRNAs | 0.972071994 | 0.000407468 |
| hsa-miR-519c-3p | selected as up-regulated miRNAs | selected as up-regulated miRNAs | 1.007102436 | 3.56E-12 |
| hsa-miR-758-5p | selected as up-regulated miRNAs | selected as up-regulated miRNAs | 0.779846301 | 0.002057688 |
| hsa-miR-10a-5p | selected as up-regulated miRNAs |  | 0.780934252 | 0.000154883 |
| hsa-miR-129-5p | selected as up-regulated miRNAs |  | 0.918782063 | 6.77E-08 |
| hsa-miR-1304-3p | selected as up-regulated miRNAs |  | 0.838356604 | 1.10E-06 |
| hsa-miR-193a-3p | selected as up-regulated miRNAs |  | 0.853664296 | 1.59E-05 |
| hsa-miR-302e | selected as up-regulated miRNAs |  | 0.673791224 | 0.010402107 |
| hsa-miR-326 | selected as up-regulated miRNAs |  | 0.780793947 | 0.0009629 |
| hsa-miR-432-5p | selected as up-regulated miRNAs |  | 0.719352983 | 7.62E-06 |
| hsa-miR-452-5p | selected as up-regulated miRNAs |  | 0.695552863 | 0.003811371 |
| hsa-miR-507 | selected as up-regulated miRNAs |  | 0.732235958 | 0.000521876 |
| hsa-miR-511-5p | selected as up-regulated miRNAs |  | 0.817863928 | 6.95E-07 |
| hsa-miR-513c-3p | selected as up-regulated miRNAs |  | 0.646211676 | 0.000263986 |
| hsa-miR-520e | selected as up-regulated miRNAs |  | 0.724187177 | 0.000151049 |
| hsa-miR-548aa | selected as up-regulated miRNAs |  | 1.00418894 | 0.000330579 |
| hsa-let-7a-5p | selected as down-regulated miRNAs | selected as down-regulated miRNAs | -3.793911018 | 2.38E-21 |
| hsa-miR-1253 | selected as down-regulated miRNAs | selected as down-regulated miRNAs | -1.60292535 | 3.76E-12 |
| hsa-miR-3144-3p | selected as down-regulated miRNAs | selected as down-regulated miRNAs | -1.819519105 | 4.95E-25 |
| hsa-miR-532-3p | selected as down-regulated miRNAs | selected as down-regulated miRNAs | -1.372006207 | 6.11E-07 |
| hsa-miR-4454 |  | selected as down-regulated miRNAs | -1.216744855 | 3.61E-64 |
| hsa-miR-519b-5p |  | selected as down-regulated miRNAs | -1.031156565 | 3.52E-08 |
