# Supplementary material for: The Possible Influence of Mediterranean Diet on Extracellular Vesicle miRNA Expression in Breast Cancer Survivors
Source: Cancers (Basel). 2020 May 26;12(6):1355. doi: 10.3390/cancers12061355 (PMC7352167; doi:10.3390/cancers12061355)
Supplement: Supplementary file 1 [file cancers-12-01355-s001.zip › Table S2.docx]

**Supplementary Table S2. Up-regulated pathway and target genes**

| Category | Term | Count | Genes | FDR |
| --- | --- | --- | --- | --- |
| GOTERM_BP_ALL | GO:2000026~regulation of multicellular organismal development | 27 | 51208, 861, 6722, 5064, 54880, 4204, 7227, 27086, 10221, 7422, 64919, 367, 5562, 694, 5728, 5600, 2735, 9253, 51684, 6608, 54206, 5335, 396, 10810, 23414, 6935, 5789 | 0.003059 |
| GOTERM_BP_ALL | GO:0045595~regulation of cell differentiation | 25 | 51208, 861, 6722, 5064, 7227, 27086, 10221, 9095, 7422, 8837, 64919, 367, 694, 5728, 5600, 2735, 9253, 51684, 9839, 6608, 54206, 396, 23414, 6935, 5789 | 0.0052693 |
| GOTERM_BP_ALL | GO:0042127~regulation of cell proliferation | 25 | 6722, 4204, 27086, 10221, 9095, 3480, 6868, 7422, 8837, 7165, 64919, 5562, 367, 694, 5728, 5600, 2735, 6608, 9180, 54206, 8314, 23414, 112399, 6935, 4953 | 0.0057672 |
| GOTERM_BP_ALL | GO:0051239~regulation of multicellular organismal process | 34 | 861, 51208, 9647, 6722, 5064, 54880, 4204, 7227, 27086, 10221, 6868, 287, 7422, 64919, 367, 5562, 694, 5728, 2735, 5600, 55914, 9253, 51684, 477, 80223, 6608, 54206, 5335, 396, 10810, 23414, 6935, 11334, 5789 | 0.0065025 |
| GOTERM_BP_ALL | GO:0045944~positive regulation of transcription from RNA polymerase II promoter | 20 | 861, 6722, 2735, 7227, 473, 27086, 84901, 9095, 9839, 2295, 6608, 148738, 7422, 64919, 3175, 29109, 367, 4772, 23414, 6935 | 0.0073856 |
| GOTERM_BP_ALL | GO:0010628~positive regulation of gene expression | 26 | 861, 6722, 9647, 4204, 7227, 27086, 9095, 55833, 2295, 287, 148738, 7422, 29109, 3175, 64919, 5562, 367, 2735, 5600, 473, 84901, 9839, 6608, 4772, 23414, 6935 | 0.0076907 |
| GOTERM_BP_ALL | GO:0008283~cell proliferation | 27 | 861, 6722, 4204, 27086, 10221, 3480, 6868, 7422, 8837, 64919, 367, 5562, 694, 5728, 5600, 2735, 9253, 599, 473, 6608, 9180, 54206, 8314, 23414, 11334, 6935, 4953 | 0.0130052 |
| GOTERM_BP_ALL | GO:0051094~positive regulation of developmental process | 20 | 861, 6722, 694, 5064, 5600, 2735, 4204, 9253, 27086, 10221, 6608, 7422, 5335, 367, 5562, 396, 10810, 23414, 6935, 5789 | 0.0141462 |
| GOTERM_BP_ALL | GO:0009888~tissue development | 26 | 861, 6722, 54880, 5782, 7227, 65985, 27086, 23657, 2295, 1844, 6868, 8837, 7422, 3175, 64919, 367, 5562, 5728, 694, 2735, 5600, 51684, 6608, 54206, 23414, 6935 | 0.0148497 |
| GOTERM_BP_ALL | GO:0009653~anatomical structure morphogenesis | 33 | 861, 6722, 5064, 54880, 4684, 7227, 214, 27086, 9095, 2295, 1844, 6868, 287, 7422, 8837, 64919, 3175, 367, 694, 5728, 2735, 9253, 226, 473, 51684, 6608, 54206, 5335, 396, 10810, 23414, 6935, 5789 | 0.0151557 |
| GOTERM_BP_ALL | GO:0050793~regulation of developmental process | 29 | 51208, 861, 6722, 5064, 54880, 4204, 7227, 27086, 10221, 7422, 8837, 64919, 367, 5562, 694, 5728, 5600, 2735, 9253, 226, 51684, 6608, 54206, 5335, 396, 10810, 23414, 6935, 5789 | 0.0163671 |
| GOTERM_MF_ALL | GO:0000981~RNA polymerase II transcription factor activity, sequence-specific DNA binding | 15 | 861, 6722, 22887, 2735, 7227, 27086, 9839, 2295, 64919, 3175, 60468, 367, 4772, 23414, 6935 | 0.0165848 |
| GOTERM_BP_ALL | GO:0040007~growth | 18 | 9647, 6722, 694, 5728, 5782, 5600, 55914, 214, 27086, 6608, 6868, 8314, 7422, 8837, 367, 51293, 57446, 23414 | 0.0259987 |
| GOTERM_BP_ALL | GO:0006357~regulation of transcription from RNA polymerase II promoter | 26 | 861, 6722, 54880, 4204, 7227, 27086, 9095, 2295, 148738, 7422, 29109, 3175, 64919, 367, 2735, 22887, 473, 51684, 84901, 9839, 6608, 60468, 4772, 23414, 112399, 6935 | 0.0319037 |
| GOTERM_BP_ALL | GO:0048523~negative regulation of cellular process | 44 | 861, 51208, 9647, 6722, 5064, 54880, 4204, 7227, 27086, 27252, 10221, 2295, 1844, 3480, 6868, 287, 148738, 8837, 7422, 64919, 3175, 367, 5562, 5728, 694, 2735, 5600, 599, 55914, 473, 51684, 477, 9839, 80223, 6608, 54206, 8314, 5335, 60468, 396, 23414, 57446, 4772, 6935 | 0.0330884 |
| GOTERM_BP_ALL | GO:0010646~regulation of cell communication | 35 | 6722, 5783, 5064, 5782, 4204, 4684, 65985, 25778, 27086, 10221, 1844, 3480, 6868, 287, 148738, 7422, 8837, 3175, 367, 5562, 5728, 2735, 5600, 599, 55914, 51684, 477, 80223, 4082, 6608, 54206, 5335, 396, 4772, 6935 | 0.0377772 |
| GOTERM_BP_ALL | GO:0010648~negative regulation of cell communication | 20 | 6722, 5728, 5064, 2735, 599, 55914, 51684, 27086, 10221, 80223, 3480, 1844, 6868, 54206, 148738, 8837, 3175, 367, 5562, 4772 | 0.0448486 |
| GOTERM_MF_ALL | GO:0005515~protein binding | 77 | 6722, 9647, 5064, 5783, 5782, 4204, 23657, 10295, 2295, 3480, 287, 6868, 148738, 8837, 63916, 55503, 7165, 2997, 23214, 5600, 55914, 51560, 9253, 473, 477, 80223, 4082, 6608, 10000, 8314, 2882, 10810, 23414, 5877, 112399, 11334, 6935, 5789, 861, 51208, 4224, 54880, 7227, 65985, 214, 27086, 10221, 27252, 311, 23199, 55833, 1844, 8487, 7422, 64919, 29109, 367, 6541, 5562, 51293, 9429, 23250, 5728, 694, 2735, 599, 226, 51684, 150864, 9839, 9180, 54206, 5335, 396, 4772, 900, 4953 | 0.0469202 |
| GOTERM_BP_ALL | GO:0023057~negative regulation of signaling | 20 | 6722, 5728, 5064, 2735, 599, 55914, 51684, 27086, 10221, 80223, 3480, 1844, 6868, 54206, 148738, 8837, 3175, 367, 5562, 4772 | 0.0469959 |
