# Supplementary material for: The Possible Influence of Mediterranean Diet on Extracellular Vesicle miRNA Expression in Breast Cancer Survivors
Source: Cancers (Basel). 2020 May 26;12(6):1355. doi: 10.3390/cancers12061355 (PMC7352167; doi:10.3390/cancers12061355)
Supplement: Supplementary file 1 [file cancers-12-01355-s001.zip › Table S3.docx]

**Supplementary Table S3. Down-regulated pathway and target genes**

| **Category** | **Term** | **Count** | **Genes** | **FDR** |
| --- | --- | --- | --- | --- |
| KEGG_PATHWAY | hsa05206:MicroRNAs in cancer | 16 | 595, 5743, 3845, 8091, 3690, 836, 7431, 1021, 3162, 4893, 993, 10642, 23405, 7057, 4609, 131405 | 1.24E-05 |
| KEGG_PATHWAY | hsa05220:Chronic myeloid leukemia | 7 | 595, 1021, 4893, 598, 3845, 7046, 4609 | 0.0375651 |
| GOTERM_BP_ALL | GO:0043603~cellular amide metabolic process | 26 | 51650, 2147, 81034, 7298, 79727, 8669, 23405, 29957, 60559, 5743, 54901, 192670, 92935, 4552, 1982, 136319, 10643, 10644, 10642, 64951, 80755, 7368, 10165, 7057, 6576, 131405 | 8.70E-05 |
| GOTERM_BP_ALL | GO:0006412~translation | 19 | 51650, 81034, 54901, 192670, 92935, 79727, 1982, 10643, 10644, 8669, 10642, 23405, 29957, 64951, 80755, 10165, 7057, 6576, 131405 | 0.0017863 |
| GOTERM_BP_ALL | GO:0042063~gliogenesis | 12 | 7431, 9289, 1021, 2147, 7099, 23405, 4771, 3845, 8091, 1814, 79727, 4609 | 0.0028104 |
| GOTERM_BP_ALL | GO:0043604~amide biosynthetic process | 20 | 51650, 81034, 54901, 192670, 92935, 79727, 1982, 10643, 10644, 8669, 10642, 23405, 29957, 64951, 80755, 7368, 10165, 7057, 6576, 131405 | 0.003035 |
| GOTERM_BP_ALL | GO:0043043~peptide biosynthetic process | 19 | 51650, 81034, 54901, 192670, 92935, 79727, 1982, 10643, 10644, 8669, 10642, 23405, 29957, 64951, 80755, 10165, 7057, 6576, 131405 | 0.003149 |
| GOTERM_BP_ALL | GO:0006518~peptide metabolic process | 21 | 51650, 2147, 81034, 54901, 192670, 92935, 79727, 1982, 10643, 10644, 8669, 10642, 23405, 29957, 64951, 80755, 60559, 10165, 7057, 6576, 131405 | 0.003607 |
| GOTERM_BP_ALL | GO:0045596~negative regulation of cell differentiation | 18 | 595, 2580, 9289, 2147, 7471, 3845, 8091, 3475, 3690, 80306, 79727, 7431, 1021, 7099, 23405, 1814, 7046, 4609 | 0.0077758 |
| GOTERM_BP_ALL | GO:0006417~regulation of translation | 13 | 7298, 192670, 79727, 1982, 136319, 10643, 8669, 10644, 23405, 10642, 80755, 7057, 131405 | 0.0151347 |
| GOTERM_BP_ALL | GO:0009314~response to radiation | 14 | 8243, 595, 598, 5743, 3845, 8091, 4734, 836, 9212, 388, 993, 1814, 2177, 4609 | 0.0221397 |
| GOTERM_BP_ALL | GO:0048608~reproductive structure development | 14 | 595, 639, 9289, 22862, 682, 598, 5743, 8091, 192670, 157506, 3880, 7812, 7046, 4609 | 0.0221397 |
| GOTERM_BP_ALL | GO:0061458~reproductive system development | 14 | 595, 639, 9289, 22862, 682, 598, 5743, 8091, 192670, 157506, 3880, 7812, 7046, 4609 | 0.0251143 |
| GOTERM_BP_ALL | GO:1901566~organonitrogen compound biosynthetic process | 26 | 51650, 81034, 7298, 79727, 54498, 535, 8669, 23405, 29957, 54901, 192670, 92935, 4552, 1982, 10643, 10644, 10642, 64951, 1814, 80755, 7368, 10165, 6576, 4609, 7057, 131405 | 0.0326799 |
| GOTERM_BP_ALL | GO:0034248~regulation of cellular amide metabolic process | 13 | 7298, 192670, 79727, 1982, 136319, 10643, 8669, 10644, 23405, 10642, 80755, 7057, 131405 | 0.0359031 |
| GOTERM_BP_ALL | GO:0007049~cell cycle | 29 | 8243, 2580, 1453, 23378, 5557, 7298, 8091, 10270, 64682, 388, 5655, 29128, 2177, 7046, 595, 598, 5743, 27243, 192670, 1982, 9212, 1021, 993, 1814, 11334, 4609, 7057, 131405, 55964 | 0.0423098 |
| GOTERM_BP_ALL | GO:0008406~gonad development | 10 | 595, 157506, 9289, 22862, 598, 7812, 8091, 192670, 7046, 4609 | 0.0498722 |
| GOTERM_CC_ALL | GO:0005737~cytoplasm | 110 | 2147, 682, 7298, 7169, 64682, 54498, 535, 8669, 3880, 5743, 3690, 4298, 79711, 1982, 4893, 83858, 6876, 1814, 1278, 10165, 11334, 4609, 10525, 8243, 79760, 822, 10423, 1453, 81034, 9904, 3845, 84191, 10270, 9910, 57410, 54935, 84705, 23589, 2177, 60559, 7046, 595, 391, 22862, 598, 192670, 10280, 7431, 136319, 7916, 1832, 134266, 23339, 993, 29099, 4771, 7812, 5432, 7057, 6576, 2580, 10241, 10857, 23378, 80306, 283377, 9415, 8773, 388, 23405, 283237, 29957, 64359, 80005, 2997, 7837, 639, 90957, 6509, 5510, 9296, 836, 4552, 157506, 9212, 51650, 7471, 4734, 79727, 1603, 55621, 11266, 27243, 54901, 6856, 3475, 3159, 92935, 3162, 1021, 10643, 10644, 7099, 10642, 52, 64951, 80755, 79922, 131405, 55964 | 7.75E-09 |
| GOTERM_CC_ALL | GO:0043227~membrane-bounded organelle | 116 | 2147, 682, 7298, 64682, 54498, 535, 3880, 29128, 5743, 3690, 4298, 79711, 4893, 83858, 1814, 1278, 10165, 11334, 4609, 10525, 8243, 79760, 822, 10423, 1453, 81034, 9904, 5557, 3845, 84191, 10270, 51616, 9910, 57410, 54935, 84705, 23589, 2177, 60559, 7046, 595, 391, 22862, 598, 5425, 81539, 192670, 10280, 7431, 7916, 136319, 1832, 134266, 23339, 993, 29099, 4771, 7812, 5432, 7057, 6576, 2580, 9289, 10241, 10857, 23378, 55589, 80306, 24138, 283377, 9415, 8773, 388, 23405, 283237, 29957, 64359, 8161, 7837, 639, 90957, 6509, 5510, 9296, 836, 4552, 157506, 9212, 8634, 51650, 7471, 8091, 4734, 1603, 79727, 55621, 23670, 11266, 27243, 54901, 6856, 3475, 3159, 92935, 3162, 1021, 10643, 10644, 7099, 10642, 10557, 52, 64951, 79922, 80755, 7368 | 4.04E-08 |
| GOTERM_CC_ALL | GO:0044444~cytoplasmic part | 93 | 2580, 2147, 10241, 682, 10857, 7298, 80306, 283377, 9415, 7169, 64682, 8773, 54498, 388, 535, 8669, 3880, 23405, 283237, 29957, 64359, 2997, 7837, 90957, 6509, 5743, 3690, 9296, 836, 4552, 1982, 157506, 9212, 83858, 4893, 1814, 1278, 10165, 11334, 4609, 8243, 10525, 79760, 51650, 822, 10423, 7471, 1453, 81034, 3845, 4734, 10270, 79727, 1603, 55621, 9910, 57410, 54935, 84705, 23589, 60559, 7046, 595, 11266, 391, 22862, 598, 27243, 54901, 6856, 3159, 192670, 92935, 10280, 7431, 136319, 3162, 1021, 134266, 10643, 23339, 10644, 993, 7099, 10642, 29099, 4771, 7812, 64951, 79922, 6576, 7057, 131405 | 4.07E-08 |
| GOTERM_CC_ALL | GO:0043231~intracellular membrane-bounded organelle | 110 | 2147, 682, 7298, 64682, 54498, 535, 29128, 5743, 3690, 4298, 79711, 4893, 83858, 1814, 1278, 10165, 11334, 4609, 10525, 8243, 79760, 822, 10423, 1453, 81034, 9904, 5557, 3845, 84191, 10270, 51616, 9910, 57410, 54935, 84705, 2177, 60559, 7046, 595, 391, 22862, 598, 5425, 192670, 10280, 7431, 7916, 136319, 1832, 134266, 23339, 993, 29099, 4771, 7812, 5432, 7057, 6576, 2580, 10241, 10857, 23378, 55589, 80306, 24138, 283377, 9415, 8773, 388, 23405, 283237, 29957, 64359, 8161, 7837, 639, 90957, 6509, 5510, 9296, 836, 4552, 157506, 9212, 8634, 51650, 7471, 8091, 4734, 79727, 1603, 55621, 11266, 27243, 54901, 6856, 3475, 3159, 92935, 3162, 1021, 10643, 10644, 7099, 10642, 10557, 64951, 80755, 79922, 7368 | 6.07E-08 |
| GOTERM_CC_ALL | GO:0043229~intracellular organelle | 115 | 2147, 682, 7298, 7169, 64682, 54498, 535, 3880, 29128, 5743, 3690, 4298, 79711, 4893, 83858, 1814, 1278, 10165, 11334, 4609, 10525, 8243, 79760, 822, 10423, 1453, 81034, 9904, 5557, 3845, 84191, 10270, 51616, 9910, 57410, 54935, 84705, 23589, 2177, 60559, 7046, 595, 391, 22862, 598, 5425, 192670, 10280, 7431, 7916, 136319, 1832, 134266, 23339, 993, 29099, 4771, 7812, 5432, 7057, 6576, 2580, 10241, 10857, 23378, 55589, 80306, 24138, 283377, 9415, 8773, 388, 23405, 283237, 29957, 64359, 8161, 7837, 639, 90957, 6509, 5510, 9296, 836, 4552, 157506, 9212, 8634, 51650, 7471, 8091, 4734, 79727, 1603, 55621, 11266, 27243, 54901, 6856, 3475, 3159, 92935, 3162, 1021, 10643, 10644, 7099, 10642, 10557, 64951, 79922, 80755, 7368, 131405, 55964 | 9.23E-08 |
| GOTERM_CC_ALL | GO:0043226~organelle | 119 | 2147, 682, 7298, 7169, 64682, 54498, 535, 3880, 29128, 5743, 3690, 4298, 79711, 4893, 83858, 1814, 1278, 10165, 11334, 4609, 10525, 8243, 79760, 822, 10423, 1453, 81034, 9904, 5557, 3845, 84191, 10270, 51616, 9910, 57410, 54935, 84705, 23589, 2177, 60559, 7046, 595, 391, 22862, 598, 5425, 81539, 192670, 10280, 7431, 7916, 136319, 1832, 134266, 23339, 993, 29099, 4771, 7812, 5432, 7057, 6576, 2580, 9289, 10241, 10857, 23378, 55589, 80306, 24138, 283377, 9415, 8773, 388, 23405, 283237, 29957, 64359, 8161, 7837, 639, 90957, 6509, 5510, 9296, 836, 4552, 157506, 9212, 8634, 51650, 7471, 8091, 4734, 1603, 79727, 55621, 23670, 11266, 27243, 54901, 6856, 3475, 3159, 92935, 1021, 3162, 10643, 10644, 7099, 10642, 10557, 52, 64951, 79922, 80755, 7368, 131405, 55964 | 3.00E-07 |
| GOTERM_CC_ALL | GO:0044424~intracellular part | 121 | 2147, 682, 7298, 7169, 64682, 54498, 535, 8669, 3880, 29128, 5743, 3690, 4298, 79711, 1982, 4893, 83858, 6876, 1814, 1278, 10165, 11334, 4609, 10525, 8243, 79760, 822, 10423, 1453, 81034, 5557, 9904, 3845, 84191, 10270, 51616, 9910, 57410, 54935, 84705, 23589, 2177, 60559, 7046, 595, 391, 22862, 598, 5425, 192670, 10280, 7431, 7916, 136319, 1832, 134266, 23339, 993, 29099, 4771, 7812, 5432, 7057, 6576, 2580, 10241, 10857, 23378, 55589, 80306, 24138, 283377, 9415, 8773, 388, 23405, 283237, 29957, 64359, 80005, 2997, 8161, 7837, 639, 90957, 6509, 5510, 9296, 836, 4552, 157506, 9212, 8634, 51650, 7471, 8091, 4734, 1603, 79727, 55621, 11266, 27243, 54901, 6856, 3475, 3159, 92935, 1021, 3162, 10643, 10644, 7099, 10642, 10557, 52, 64951, 79922, 80755, 7368, 131405, 55964 | 1.47E-06 |
| GOTERM_CC_ALL | GO:0005622~intracellular | 122 | 2147, 682, 7298, 7169, 64682, 54498, 535, 8669, 3880, 29128, 5743, 3690, 4298, 79711, 1982, 4893, 83858, 6876, 1814, 1278, 10165, 11334, 4609, 10525, 8243, 79760, 822, 10423, 1453, 81034, 5557, 9904, 3845, 84191, 10270, 51616, 9910, 57410, 54935, 84705, 23589, 2177, 60559, 7046, 595, 391, 22862, 598, 5425, 192670, 10280, 7431, 7916, 136319, 1832, 134266, 23339, 993, 29099, 4771, 7812, 5432, 7057, 6576, 2580, 9289, 10241, 10857, 23378, 55589, 80306, 24138, 283377, 9415, 8773, 388, 23405, 283237, 29957, 64359, 80005, 2997, 8161, 7837, 639, 90957, 6509, 5510, 9296, 836, 4552, 157506, 9212, 8634, 51650, 7471, 8091, 4734, 1603, 79727, 55621, 11266, 27243, 54901, 6856, 3475, 3159, 92935, 1021, 3162, 10643, 10644, 7099, 10642, 10557, 52, 64951, 79922, 80755, 7368, 131405, 55964 | 2.92E-06 |
| GOTERM_CC_ALL | GO:0044446~intracellular organelle part | 86 | 2147, 10241, 682, 10857, 7298, 23378, 55589, 80306, 9415, 7169, 64682, 8773, 388, 535, 3880, 23405, 283237, 29128, 29957, 8161, 639, 6509, 5743, 3690, 4298, 9296, 836, 79711, 4552, 157506, 9212, 83858, 4893, 8634, 1278, 10165, 4609, 8243, 10525, 79760, 51650, 822, 10423, 7471, 1453, 81034, 9904, 5557, 84191, 8091, 4734, 10270, 79727, 1603, 55621, 51616, 57410, 2177, 60559, 595, 391, 22862, 598, 5425, 27243, 54901, 6856, 3159, 92935, 10280, 7431, 136319, 1832, 3162, 1021, 134266, 23339, 993, 7099, 10557, 4771, 7812, 64951, 5432, 7057, 6576 | 0.0002839 |
| GOTERM_CC_ALL | GO:0044422~organelle part | 86 | 2147, 10241, 682, 10857, 7298, 23378, 55589, 80306, 9415, 7169, 64682, 8773, 388, 535, 3880, 23405, 283237, 29128, 29957, 8161, 639, 6509, 5743, 3690, 4298, 9296, 836, 79711, 4552, 157506, 9212, 83858, 4893, 8634, 1278, 10165, 4609, 8243, 10525, 79760, 51650, 822, 10423, 7471, 1453, 81034, 9904, 5557, 84191, 8091, 4734, 10270, 79727, 1603, 55621, 51616, 57410, 2177, 60559, 595, 391, 22862, 598, 5425, 27243, 54901, 6856, 3159, 92935, 10280, 7431, 136319, 1832, 3162, 1021, 134266, 23339, 993, 7099, 10557, 4771, 7812, 64951, 5432, 7057, 6576 | 0.0009013 |
| GOTERM_CC_ALL | GO:0005912~adherens junction | 17 | 10525, 2580, 391, 822, 1453, 682, 3845, 3690, 3159, 1982, 7431, 1832, 8773, 388, 57410, 4771, 23670 | 0.0303252 |
| GOTERM_CC_ALL | GO:0070161~anchoring junction | 17 | 10525, 2580, 391, 822, 1453, 682, 3845, 3690, 3159, 1982, 7431, 1832, 8773, 388, 57410, 4771, 23670 | 0.0406708 |
| GOTERM_CC_ALL | GO:0044446~intracellular organelle part | 86 | 2147, 10241, 682, 10857, 7298, 23378, 55589, 80306, 9415, 7169, 64682, 8773, 388, 535, 3880, 23405, 283237, 29128, 29957, 8161, 639, 6509, 5743, 3690, 4298, 9296, 836, 79711, 4552, 157506, 9212, 83858, 4893, 8634, 1278, 10165, 4609, 8243, 10525, 79760, 51650, 822, 10423, 7471, 1453, 81034, 9904, 5557, 84191, 8091, 4734, 10270, 79727, 1603, 55621, 51616, 57410, 2177, 60559, 595, 391, 22862, 598, 5425, 27243, 54901, 6856, 3159, 92935, 10280, 7431, 136319, 1832, 3162, 1021, 134266, 23339, 993, 7099, 10557, 4771, 7812, 64951, 5432, 7057, 6576 | 0.0002839 |
| GOTERM_CC_ALL | GO:0044422~organelle part | 86 | 2147, 10241, 682, 10857, 7298, 23378, 55589, 80306, 9415, 7169, 64682, 8773, 388, 535, 3880, 23405, 283237, 29128, 29957, 8161, 639, 6509, 5743, 3690, 4298, 9296, 836, 79711, 4552, 157506, 9212, 83858, 4893, 8634, 1278, 10165, 4609, 8243, 10525, 79760, 51650, 822, 10423, 7471, 1453, 81034, 9904, 5557, 84191, 8091, 4734, 10270, 79727, 1603, 55621, 51616, 57410, 2177, 60559, 595, 391, 22862, 598, 5425, 27243, 54901, 6856, 3159, 92935, 10280, 7431, 136319, 1832, 3162, 1021, 134266, 23339, 993, 7099, 10557, 4771, 7812, 64951, 5432, 7057, 6576 | 0.0009013 |
| GOTERM_CC_ALL | GO:0005912~adherens junction | 17 | 10525, 2580, 391, 822, 1453, 682, 3845, 3690, 3159, 1982, 7431, 1832, 8773, 388, 57410, 4771, 23670 | 0.0303252 |
| GOTERM_CC_ALL | GO:0070161~anchoring junction | 17 | 10525, 2580, 391, 822, 1453, 682, 3845, 3690, 3159, 1982, 7431, 1832, 8773, 388, 57410, 4771, 23670 | 0.0406708 |
